# Can Global Variation of Nasopharynx Cancer Be Retrieved from the Combined Analyses of IARC Cancer Information (CIN) Databases?

**DOI:** 10.1371/journal.pone.0022039

**Published:** 2011-07-07

**Authors:** Xin Sun, Li-Ping Tong, Yu-Tong Wang, Yong-Xiang Wu, Hong-Shen Sheng, Lian-Jun Lu, Wen Wang

**Affiliations:** 1 Department of Paediatrics, Xi'jing Hospital, Fourth Military Medical University, Xi'an, Shaanxi Province, People's Republic of China; 2 Department of Teaching and Training, Xi'jing Hospital, Fourth Military Medical University, Xi'an, Shaanxi Province, People's Republic of China; 3 Department of Emergency, Xi'jing Hospital, Fourth Military Medical University, Xi'an, Shaanxi Province, People's Republic of China; 4 Department of Otolaryngology and Head Neck Surgery, Xi'jing Hospital, Fourth Military Medical University, Xi'an, Shaanxi Province, People's Republic of China; 5 Department of Otolaryngology, General Hospital of Chengdu Military Area, Tian Hui Town, Sichuan Province, People's Republic of China; 6 Unit for Evidence Based Medicine, K. K. Leung Brain Research Centre, Fourth Military Medical University, Xi'an, Shaanxi Province, People's Republic of China; Harvard Medical School, United States of America

## Abstract

**Background:**

The international nasopharynx cancer (NPC) burdens are masked due to the lack of integrated studies that examine epidemiological data based on up-to-date international disease databases such as the Cancer Information (CIN) databases provided by the International Agency for Research on Cancer (IARC).

**Methods:**

By analyzing the most recently updated NPC epidemiological data available from IARC, we tried to retrieve the worldwide NPC burden and patterns from combined analysis with GLOBOCAN2008 and the Cancer Incidence in Five Continents (CI5) databases. We provide age-standardized rates (ASR) for NPC mortality in 20 highest cancer registries from GLOBOCAN2008 and the World Health Organization (WHO) mortality databases, respectively. However, NPC incidence data can not be retrieved since it is not individually listed in CI5 database. The trend of NPC mortality was investigated with Joinpoint analysis in the selected countries/regions with high ASR.

**Results:**

GLOBOCAN 2008 revealed that the highest NPC incidence rates in 2008 were in registries from South-Eastern Asia, Micronesia and Southern Africa with Malaysia, Indonesia and Singapore ranking the top 3. WHO mortality database analysis revealed that China Hong Kong, Singapore and Malta ranks the top 3 regions with the highest 5-year mortality rates.

**Conclusions:**

NPC mortality rate is about 2–3 times higher in male than that in female, and shows decrease tendency in those selected countries/regions during the analyzed periods. However, the integrated analyses of the current IARC CIN databases may not be suitable to retrieve epidemiological data of NPC. Much effort is required to improve the local cancer entry and regional death-reporting systems so as to aid similar studies.

## Introduction

Nasopharyngeal cancer (NPC) is a specific cancer with ethnic and geographic distributions; its etiology is far from being completely understood. NPC is rare in most parts of the world, where incidences of age standardized rates (ASR) are generally below 1 per 100,000 person-years [1], [2]. However, in some well-defined populations, including natives of southern China, Southeast Asia, the Arctic and the Middle East/North Africa, NPC is a leading form of cancer [1], [2], [3]. The distinctive racial/ethnic and geographic distribution of NPC suggests that both environmental factors and genetic traits contribute to its development. To elucidate their impacts on the incidence as well as mortality, it is necessary to figure out the worldwide tendency of NPC. However, the international variations in NPC are masked because of the lack of integrated studies that examine not only incidence but also mortality data based on up-to-date international disease database. Thus, we tried to describe the global incidence and mortality patterns of NPC by using the most recent data available from the International Agency for Research on Cancer (IARC).

IARC is part of the World Health Organization (WHO). Various databases containing information on the global occurrence of cancer were held and managed by the Section of Cancer Information (CIN) of IARC and can be obtained via the website of “CANCERMondial (http://www-dep.iarc.fr/)”. The CIN databases available for the current study include: GLOBOCAN, Cancer Incidence in Five Continents (CI5) and WHO mortality databases. Each database covers some specific aspects and an integrated view of a specific cancer is expected to be achieved via combined analyses of these databases. Furthermore, these data can be abstracted for off-line analysis as well as being analyzed with IARC's online analysis tools. GLOBOCAN2008 provides access to the most recent estimates (for 2008) of the incidence of, and mortality from 28 major cancers all over the world [4]. CI5 provides access to detailed information on the incidence of cancer recorded by global cancer registries (regional or national). WHO mortality database presents long time series of selected cancer mortality recorded in selected countries. The world-wide tendencies for rectal cancer has been investigated based on the integrated analyses of these databases, which confirmed their usefulness in similar study [5].

As a specific cancer type with racial/ethnic and geographic distribution, we had expected that the worldwide variation of NPC can be investigated via the integrated analyses of these databases. But there are differences in the number of data entries, recording periods, and cancer classifying protocols among 3 databases, which can significantly affect the analytic output. For example, the code for NPC according to the International Classification of Diseases for Oncology (ICD-O) is C11. In CI5, this code is not individually listed, thus, we could not use CI5 database to retrieve incidence rate and tendency during the observing period. However, in GLOBOCAN2008 and WHO mortality databases, C11 is individually listed so that estimation of mortality rate and tendency can be achieved.

Based on the above-mentioned limitations, we performed the current study for two purposes: first, whether the integrated analyses of IARC CIN databases can be used to provide the global variations of NPC; second, if yes, how accurate is the output and what can be improved to get more accurate output.

## Methods

National level contemporary estimates of the incidence of, and mortality from NPC are collected from GLOBOCAN2008 project that presents latest data for 2008. Cancer incidence data are compiled and provided by IARC in volumes I to IX of CI5 by world-wide population-based cancer registries at the national or regional levels. The most recent volume of CI5 (volume IX) includes data from 300 registries in 61 countries. We used data from volume IX of CI5 and tried to display cross-sectional, aggregated NPC incidence rates for 1998–2002 for most of the select registries. The mortality data were abstracted from the death certificates that were compiled in a WHO mortality database. The code for NPC according to the International Classification of Diseases for Oncology (ICD-O) is C11. In CI5, this code is not individually listed, thus we selected the C09-14 (pharynx) to approximately represent NPC, while in WHO and GLOBOCAN2008 databases, C11 was individually listed to represent NPC. C09-14 covers all types of pharyngeal cancer that differs greatly in incidence, risk factors, management and prognosis. Since there is no study supporting the extrapolation of NPC data from pharyngeal cancer, we failed to retrieve NPC incidence data from CI5 database.

For epidemiological and preventive study of medicine, the tendency of disease incidence and mortality rate are very important. Joinpoint regression analysis is commonly used for determining the tendency of disease incidence/mortality based on the single-year incidence/mortality data with fitting a series of joined straight lines on a logarithmic scale to the trends in the annual ASRs annual change [6]. Long time recorded data among the top 20 countries with the highest mortality rate are abstracted from WHO mortality database for Joinpoint analysis. Joinpoint analysis output were expressed as annual percent change (APC) (ie, the slope of the line segment) [6] and terms “increase” or “decrease” were used when the APC was statistically significant (P<.05); otherwise the term “stable” was used. Due to the variability in the economical development and completeness of health recording system, the quality of long-time recorded data abstracted from WHO mortality database scored differently, thus the reliability of tendency analysis varies among NPC entries.

## Results

### Global differences of NPC incidences

#### 1.1 GLOBOCAN2008 database

**Figure 1:**
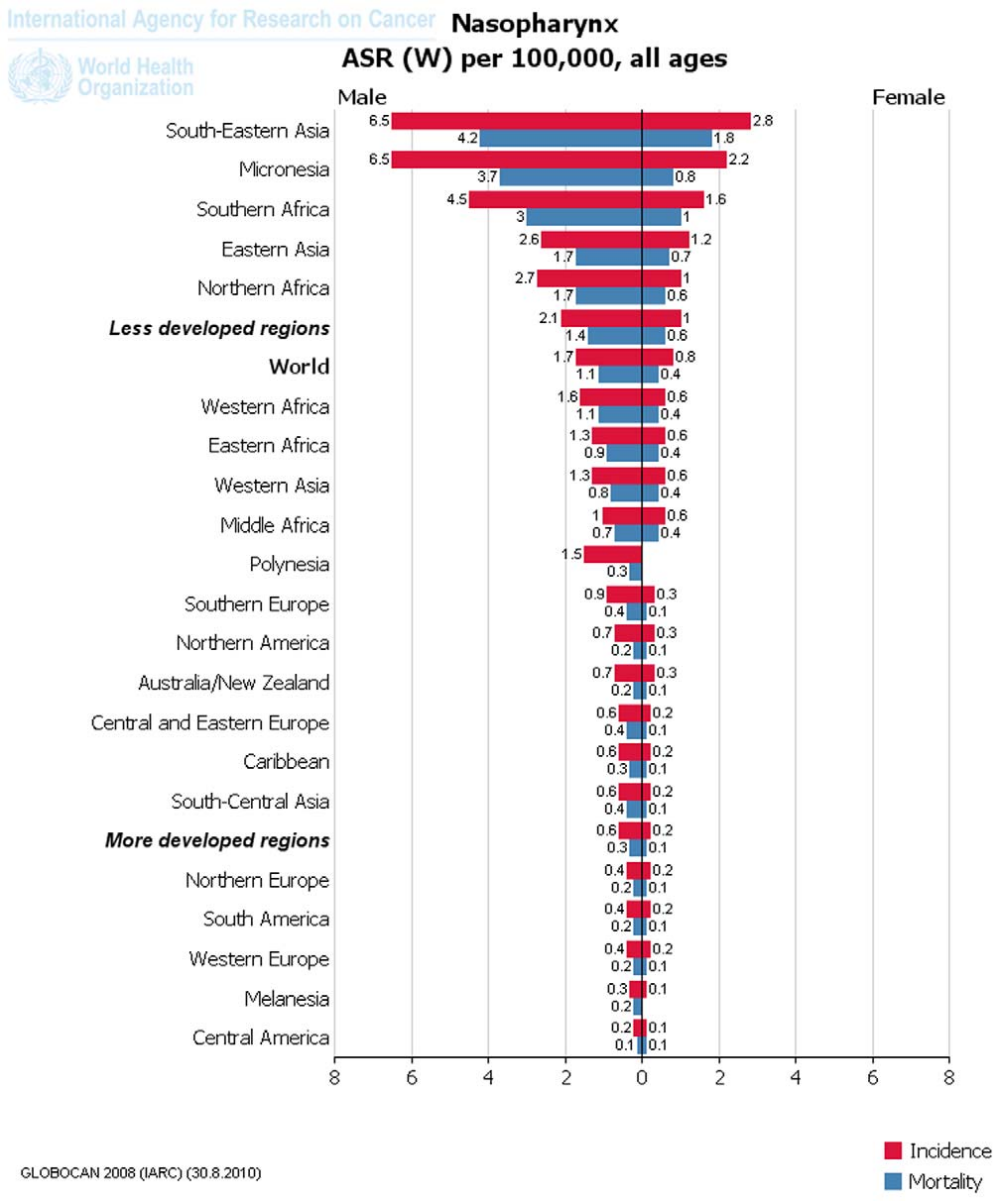
Continental incidence (red) and mortality (blue) of NPC demonstrated as ASR in 100,000 people-years from GLOBOCAN2008 database.

**Figure 2:**
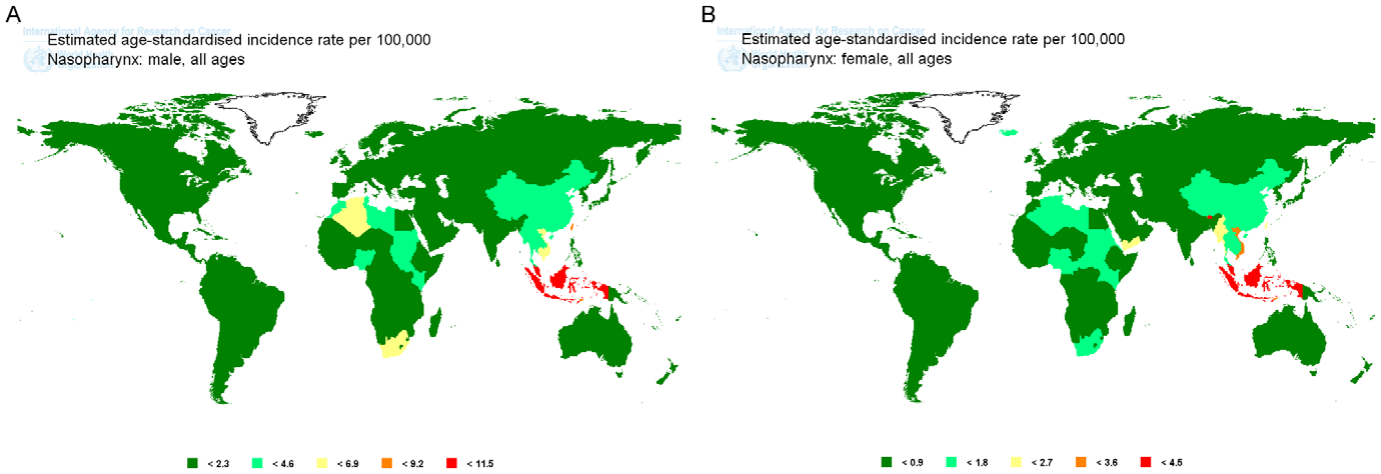
GLOBOCAN2008 map for global NPC incidence in male (A) and female (B). Demonstrated as ASR per 100,000 people-years.

**Figure 3:**
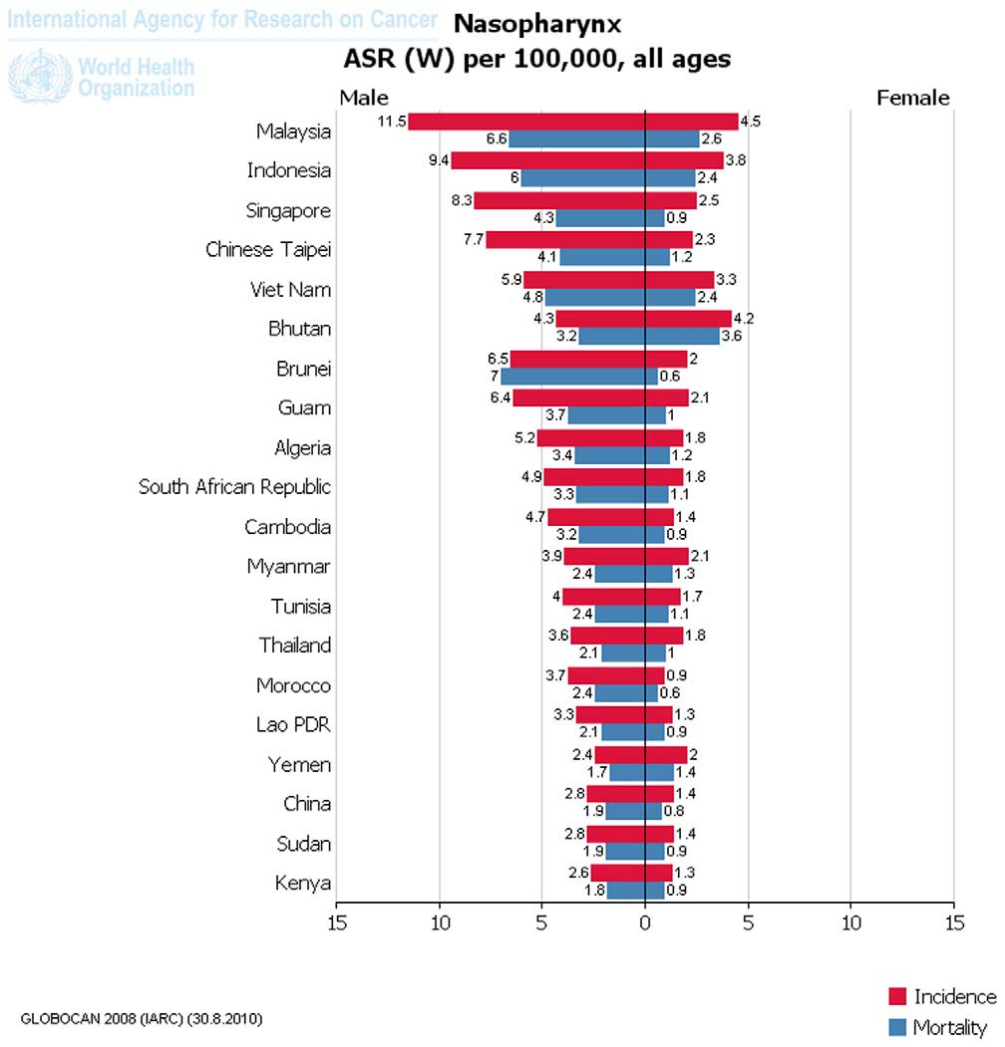
National incidence (red) and mortality (blue) of NPC demonstrated as ASR per 100,000 people-years from GLOBOCAN2008 database.

Based on the analysis of 2008 data from GLOBOCAN2008 database, the estimated NPC incidence rates vary markedly worldwide, with ASRs among males reported to range from 0.2 in Central America to 6.5 per 100,000 person-years in South-Eastern Asia. The majority of registries with the highest incidence rates of NPC in male were located in Asia, Micronesia and South Africa. In contrast, the lowest rates were observed from registries in Central America, Melanesia, Western Europe, South America and North Europe (**Fig. 1** **,** **Fig. 2A**). At the national level, NPC incidence rates among males in Malaysia (11.5), Indonesia (9.4), Singapore (8.3), Taipei of China (7.7), Brunei (6.5), Guam (6.4), Viet Nam (5.9), Algeria (5.2), South African Republic (4.9), Cambodia (4.7), Bhutan (4.3), Tunisia (4), Myanmar (3.9), Morocco (3.7), Thailand (3.6), Lao PDR (3.3), China (2.8), Sudan (2.8), Kenya (2.6) and Yemen (2.4) rank top 20 all over the world (**Fig. 3**).

When looking at the case for female population, NPC incidence rates were reported to range from 0.1 in Central America to 2.8 in South-Eastern Asia. The majority of registries with the highest estimations for NPC in female were located in Asia, Micronesia and South Africa. In contrast, the lowest rates were observed from registries in Central America, Melanesia, Western Europe, South America and North Europe (**Fig. 1** **,** **Fig. 2B**). At the national level, NPC incidence rates among females in Malaysia (4.5), Bhutan (4.2), Indonesia (3.8), Viet Nam (3.3), Singapore (2.5), Taipei of China (2.3), Guam (2.1), Myanmar (2.1), Brunei (2), Yemen (2), Algeria (1.8), South African Republic (1.8), Thailand (1.8), Tunisia (1.7), Cambodia (1.4), China (1.4), Sudan (1.4), Lao PDR (1.3), Kenya (1.3) and Morocco (0.9) rank top 20 all over the world (**Fig. 3**).

These data reveal that NPC favors male more than female in that ASRs are twice as high in males as those in females. On the other hand, the low NPC incidence rate in both male and female Chinese populations might be the result of dilution by populations from other registries with low incidence rate. Thus, to look into more details, the regional registry data are required.

#### 1.2 CI5 database can not be used to retrieve 5-year NPC incidence rate

However, NPC (C11) is not individually listed in the current CI5 IX database; the NPC regional incidence rates can not be retrieved.

The fact that there is no individual category for NPC in CI5 database might reflect that the NPC incidence rates are too low in most global regions (even in regions of China other than Hong Kong, Taipei and Guangzhou) to be reported. The health caring systems in the high incidence regions (majorly in Chinese population of the developing countries) are not good enough to cover the epidemiological monitoring of NPC. On the other hand, although there is an individual category for NPC in GLOBOCAN2008 database, it may not reflect the real NPC epidemiology based on two reasons: first, most of the entries for GLOBOCAN2008 database are at the national levels, while for the case of NPC, it has a significant geographic distribution; second, the output of GLOBOCAN2008 is based on the estimation from the current available literature and resources, it is not a real incidence rate.

#### 1.3 NPC incidence tendency can not be estimated from CI5 plus database

It would be nice for the prevention purpose if we can obtain the tendency of NPC incidence. CI5 Plus database offered annual incidence rate record of some cancers in some entries and made the tendency analysis with Joinpoint software possible. Unfortunately, since there is no individual category for NPC (NPC is cover by an even wider range C00-14) in this database and most of the high incidence rate entries are not recorded, we could not analyze the accurate tendency of NPC incidence rate during the past years.

The fact that individual category for NPC (C11) is not listed separately in CI5 plus database reflect the reality: 1. NPC is not prevalent in those entries with well developed cancer registry systems; 2. Some NPC high incidence entries fails to report the annual NPC incidence rate because of the less developed cancer registry systems. For example, Zhongshang and Sihui of Guangdong province, China are two entries with high NPC incidence rate [7], unfortunately, they haven't been included in the WHO database system and we could not track the NPC tendency there by using the current CI5 plus database. At the current stage, CI5 plus database might be very helpful to investigate the global tendency of some cancer, such as rectal cancer or lung cancer that is top health threatening to world population, or some cancer with a well developed international cancer registry system. Developing countries like China need to put much more effort in improving the cancer registry system.

### Global differences of NPC mortality

Another parameter for cancer epidemiological study is mortality. Since there is specific category for NPC in both GLOBOCAN2008 and WHO mortality databases, we compared the most recent estimated annual (from GLOBOCAN2008 database) and real mortalities in 5-year period (from WHO mortality database).

#### 2.1 GLOBOCAN2008 database

**Figure 4:**
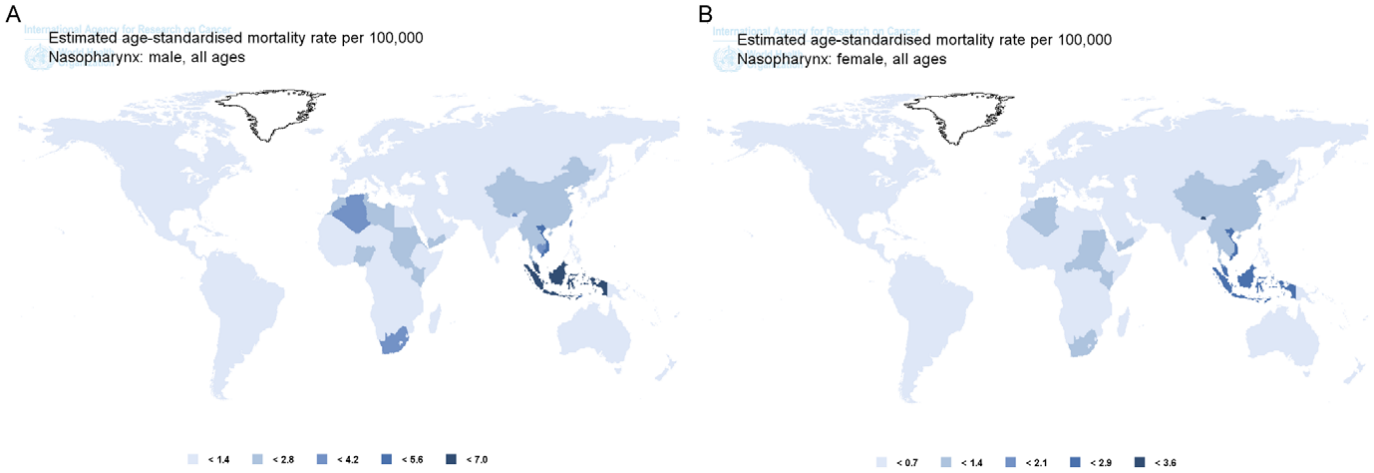
GLOBOCAN2008 map for global NPC mortality rate in male (A) and female (B). Demonstrated as ASR per 100,000 people-years.

In GLOBOCAN2008 database, data under the category of “nasopharynx cancer (C11)” were collected. NPC mortality rates vary markedly worldwide, with rates per **100,000** person years among males reported to range from 0.1 in Central America to 4.2 in South-Eastern Asia. The majority of registries with the highest incidence rates of NPC in male were located in Asia, Micronesia and South Africa. In contrast, the lowest rates were observed from registries in Central America, Melanesia, Western Europe, South America and North Europe (**Fig. 1** **,** **Fig. 4A**). At the national level, NPC mortality rates among males in Brunei (7), Malaysia (6.6), Indonesia (6), Viet Nam (4.8), Singapore (4.3), Taipei (4.1, China), Guam (3.7), Algeria (3.4), South African Republic (3.3), Cambodia (3.2), Bhutan (3.2), Tunisia (2.4), Myanmar (2.4), Morocco (2.4), Thailand (2.1), Lao PDR (2.1), China (1.9), Sudan (1.9), Kenya (1.6) and Yemen (1.7) rank top 20 all over the world (**Fig. 3**).

NPC mortality rates in female were reported to range from 0.1 in Central America to 1.8 in South-Eastern Asia. The majority of registries with the highest mortality rates of NPC in female were located in Asia, Micronesia and South Africa. In contrast, the lowest rates were observed from registries in Central America, Melanesia, Western Europe, South America and North Europe (**Fig. 1** **,** **Fig. 4B**). At the national level, NPC mortality rates among female in Bhutan (3.6), Malaysia (2.6), Indonesia (2.4), Viet Nam (2.4), Yemen (1.4), Myanmar (1.3), Taipei (1.2, China), Algeria (1.2), South African Republic (1.1), Tunisia (1.1), Guam (1), Thailand (1), Cambodia (0.9), Singapore (0.9), Sudan (0.9), Lao PDR (0.9), Kenya (0.9), China (0.8), Brunei (0.6) and Morocco (0.6) rank top 20 all over the world (**Fig. 3**).

#### 2.2 WHO database (1998–2002)

**Figure 5:**
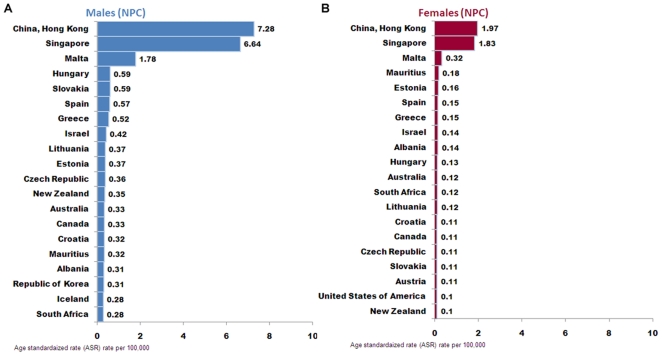
Regional mortality of NPC (C11) demonstrated as ASR from WHO mortality database of male (A) and female (B) populations.

In WHO mortality database, data under the category of “nasopharynx cancer (C11)” from 1998–2002 were collected. The analysis output showed that the entries with top 20 mortality rate in male were Hong Kong (7.28, China), Singapore (6.64), Malta (1.78), Hungary (0.59), Slovakia (0.59), Spain (0.57), Greece (0.52), Israel (0.42), Lithuania (0.37), Estonia (0.37), Czech Republic (0.36), New Zealand (0.35), Australia (0.33), Canada (0.33), Croatia (0.32), Mauritius (0.32), Albania (0.31), Republic of Korea (0.31), Iceland (0.28) and South Africa (0.28) (**Fig. 5A**). The entries with top 20 mortality rate in female were Hong Kong (1.97, China), Singapore (1.83), Malta (.32), Mauritius (0.18), Estonia (0.16), Spain (0.15), Greece (0.15), Israel (0.14), Albania (0.14), Hungary (0.13), Australia (0.12), South Africa (0.12), Lithuania (0.12), Croatia (0.11), Canada (0.11), Czech Republic (0.11), Slovakia (0.11), Austria (0.11), Unite States of America (0. 1) and New Zealand (0.1) (**Fig. 5B**).

When comparing the top-20 mortality rate entries output from GLOBOCAN2008 and WHO mortality databases, the rank and ASR values are not consistent. This is because first, the entries for each database are not same: WHO database includes regional entries below national level which covers some NPC high incidence areas like Hong Kong; the entry for GLOBOCAN2008 are basically at nation levels that may dilute the high mortality rate in a specific region within a country (especially within China). Second, the calculation protocols used in each database are not same: GLOBOCAN2008 provides the annual mortality rate estimation of 2008 while WHO database provides the mortality rate during 1998–2002. Although differences exist, these two database outputs together suggested the geographical feature of NPC that NPC mortality rates are high in south East Asia, especially some Chinese population in regions like Hong Kong and Singapore.

#### 2.3 NPC mortality rate trends: Joinpoint analyses of the available WHO data

Along with the advance of cancer therapy, we expect that the mortality rate will gradually decrease. But this is not always true for some specific cancers (eg. brain and central nervous system cancers). In WHO mortality database, the annual NPC mortality rate records are available for some selected entries, and the Joinpoint analysis can be performed over them to determine the mortality tendency. Since insufficient women data of annual mortality rate can be obtained from WHO database for Joinpoint analysis, we tried to abstract available data from male population in 14 out of the top 20 registries and checked their NPC mortality tendencies. According to the literature, the NPC mortality rate is several times higher in old than in young male population [7], thus we performed Joinpoint analysis from the all age population and that aged older than 30 years.

Mortality trend analyses for 14 entries demonstrated that male NPC mortality decreased in 8, increased in 1 and kept stable in 5 entries (**Table 1**). In China Hong Kong, one high incidence region, mortality rate decrease by 1.5% and 3.6% per year during the periods of 1966–1983 and 1983–2007, respectively. However, in another high incidence region, Singapore, mortality rate increased by 4.2% per year from 1966 through 1976 and then decrease by 1.9% and 6.1% per year during the periods of 1976–1996 and 1996–2006, respectively.

**Table 1:** NPC mortality rate trends in all age male population with Joinpoint analyses on the available data of the 14 out of the top 20 mortality rate registries.

	Trend 1		Trend 2		Trend 3	
	years	APC [95% CI]	years	APC [95% CI]	years	APC [95% CI]
0–85+ years						
**China Hong Kong**	1966–1983	−1.5*[−2.4 – −0.68]	1983–2007	−3.6* [−4.1 – −3.2]		
**Singapore**	1966–1976	4.2* [0.7 – 7.8]	1976–1996	−1.9* [−2.9 – −0.9]	1996–2006	−6.1* [−8.4 – −3.7]
**Malta**	1978–2007	−2.3 [−4.6 – 0.1]	1999–2007	−10.1 [−24.0 – 6.4]		
**Slovakia**	1992–2005	−2.9* [−5.7 – −0.1]				
**Greece**	1966–2008	2.2* [1.5 – 2.9]				
**Isreal**	1975–2006	−1.4* [−2.7 – −0.1]				
**Lithuania**	1993–2008	2.2 [−2.1 – 6.7]				
**Czech Republic**	1986–2008	−2.3* [−3.8 – −0.8]				
**Australia**	1955–1990	2.6* [1.7 – 3.5]	1990–2006	−4.5* [−6.3 – −2.6]		
**Canada**	1955–1988	1.9* [1.3 – 2.5]	1988–2004	−2.6* [−4.0 – −1.3]		
**Croatia**	1985–2008	−2.4 [−5.0 – 0.3]				
**Albania**	1987–2004	−6.5* [−9.9 – −3.0]				
**South Africa**	1993–2006	−0.3 [−4.7 – 4.3]				
**USA**	1960–1998	−0.8* [−1.1 – −0.5]	1998–1999	−31.1 [−– −]	1999–2005	−0.0 [−3.9 – 4.1]

*The average APC is statistically significant from zero.

Canada and Australia show first increase (by 1.9% per year during 1955–1988 in Canada and 2.6% per year during 1955–1990 in Australia) and then decrease (by 2.6% per year during 1988–2004 in Canada and 4.5% per year during 1990–2006 in Australia). The increase tendency probably can be explained by the Chinese immigration during this period. These two countries, with relatively small populations, are among the popular immigrating countries for Chinese. Before late 1990s, the immigrated Chinese majorly from south-east China region with high NPC incidence such as Hong Kong, Taipei and Guangzhou. The gradually increased mortality rate might be the “imported” effect. While, after the late 1990s, along with the Chinese reforming and opening policy, more Chinese from NPC low incidence region move to these two countries. At the same time, with the changed environment and changed eating habit, descendents from the first generation Chinese immigrants were supposed to have a decreased NPC incidence and mortality rates. Thus, the decreased tendency in these two countries can be observed in the past 2 decades. To confirm this hypothesis, more detailed population studies in these countries are needed.

Greece is the only one country showed constant increase of NPC mortality rate by 2.2% per year from 1966 through 2008. While, when taken the ASR from each entry into consideration (0.52 ASR per 100,000 person years in Greece), the increase may not explain anything. The situation in Greece needs more observation.

When Joinpoint analysis was performed in the population of older than 30 years, similar tendencies for each entry can be observed (**Table 2**). This supported that the NPC mortality tendency was majorly contributed by this population group with age older than 30 years.

**Table 2:** NPC mortality rate trends in population older than 30 years with Joinpoint analyses on the available data of the 14 out of the top 20 mortality rate registries.

	Trend 1		Trend 2		Trend 3	
	years	APC [95% CI]	years	APC [95% CI]	years	APC [95% CI]
**China Hong Kong**	1966–1983	−1.4*[−2.3 – −0.5]	1983–2007	−3.6* [−4.0 – −3.2]		
**Singapore**	1966–1975	4.9* [−0.8 – 9.1]	1975–1995	−1.6* [−2.6 – −0.6]	1995–2006	−5.8* [−7.7 – −3.8]
**Malta**	1978–2007	−2.3 [−4.6– −0.1]				
**Slovakia**	1992–2005	−2.9 [−6.3 – 0.5]				
**Greece**	1966–2008	2.0* [1.3 – 2.7]				
**Isreal**	1975–2006	−1.4* [−2.7 – −0.1]				
**Lithuania**	1993–2008	1.8 [−2.9 – 6.6]				
**Czech Republic**	1986–2008	−2.2* [−3.7 – −0.6]				
**Australia**	1955–1992	2.6* [1.9– 3.4]	1992–2006	−6. 1* [−8.4– −3.8]		
**Canada**	1955–1988	1.8* [1.0 – 2.5]	1988–2004	−2.1* [−3.6 – −0.6]		
**Croatia**	1985–2008	−2.4 [−4.9 – 0.2]				
**Albania**	1987–2004	−7.4* [−10.7 – −4.0]				
**South Africa**	1994–2006	1.2 [−2.8 – 5.4]				
**USA**	1960–1983	−0.1 [−0.6 – 0.4]	1983–2005	−1.8[−2.2 – −1.4]		

*The average APC is statistically significant from zero.

**Figure 6:**
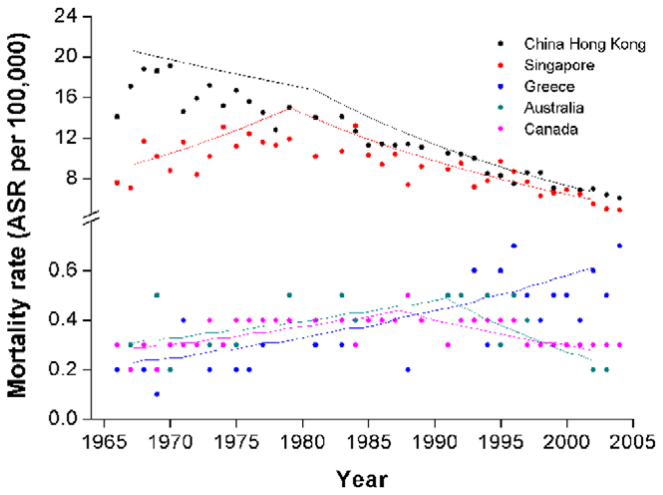
Representative joint point analysis output from NPC mortality data of China Hong Kong, Singapore, Greece, Australia and Canada. Dots represent observed value and lines represent modeled data. In this analysis, two join points were set.

In our analysis, the available entry period of WHO mortality database varied from 12 to 50 years (median 29 yr), it is highly possible that a shorter entry period may not reveal the real tendency. To check the tendency within a fixed long period, we selected 5 entries with longer entry period that represent decreased tendency with high (China Hong Kong and Singapore) or low (Australia and Canada) morality rate as well as increased tendency (Greece). After matching their entry periods from 1966 to 2004, the Joinpoint analysis was performed. This further analysis did not change the conclusions obtained by previous analysis and the curves for Joinpoint analysis from these entries were offered as examples (**Fig. 6**).

## Discussion

Based on the integrated analyses of GLOBOCAN and WHO databases, we described that NPC showed a racial/ethnic and geographic distribution that South-east Asia, especially Hong Kong and Guangdong province of China are with highest incidence and mortality rates. During the past several decades, the mortality rates of the top 20 ranked regions showed decrease tendencies. However, since NPC is not individually list in CI5 and CI5 plus databases, we failed to retrieve the 5-year NPC incidence and incidence tendency, respectively. Due to the incomplete cancer entries in those NPC high incidence regions (for all three databases), cautions should be put when explain our data.

### 1. Varied database quality limited the high quality output

#### 1.1. NPC accounts for the majority of pharynx cancers in high incidence rate regions

Since incidence rates of pharynx cancer (C09-14) instead of NPC (C11) were collected from CI5 and CI5 plus databases, one very important issue is to address whether these incidence rates of pharynx cancer can reflect NPC situation. By comparing the values obtained from CI5 (**Figure S1**) or GLOBOCAN2008 databases with epidemiological data from individual regions, we found that incidence output from CI5 and GLOBOCAN2008 databases are quite similar with regional reports from the high incidence region such as Malaysia [8], Singapore [9], [10], China Hong Kong [10], [11], etc. But this is not true for some countries/regions that are known to have a low NPC incidence rates such as France [12] and Australia [13]. For example, according to the CI5 output, several regions from France rank within top 10 highest incidence rates in case of pharynx cancer, while this is not true when we obtained data for NPC itself from GLOBOCAN2008. These finding confirmed that the geographic distribution of NPC: in those high incidence rate regions, NPC accounts for the majority of pharynx cancer; while in those low incidence rate regions, NPC accounts for a very little portion of pharynx cancer.

#### 1.2 The continuous declines in NPC incidence and mortality rates and possible reasons

NPC is not a disease of modern environmental hazards since ancient China [14] and Egypt [15] had already demonstrated higher incidence and mortality; it is rather, closely related with genetic and/or stable environmental risk factors that may have persisted for centuries. According to modern cancer registry data, NPC incidence has remained high in Southeast Asia for several decades [16], [17]. However, significant steady declines in incidence and mortality has occured in Hong Kong since the 1970s [11], [16], in Taiwan since the 1980s [17], and in Singapore Chinese since the late 1990s [1], [2]. Such a drastic change may be attributable to the onset of rapid economic development, which occurred in the mid-1940s in Hong Kong, the 1950s in Taiwan, and the 1960s in Singapore [14]. On the other hand, the incidence rate of NPC increased among Singapore Malays between 1968 and 1997 [18], and remained steady or increased slightly (among males in Cangwu county of Guangzhou city) in Southeastern China between 1978/1983 and 2002 [7].

#### 1.3 Limitations of the current findings

The analysis from GLOBOCAN database suggested that the highest incidence rates are in the southeast of China. In almost all populations surveyed, the incidence of NPC is 2- to 3-fold higher in males than in females and this is consistent with previous report [1]. However, the current findings were limited majorly because of the varied database qualities in cancer entry systems. Highest NPC incidence has been reported among people of Guangdong province and Guangxi region of China where the incidence of NPC reaches 50 or more per 100000 person years [19]. Unfortunately, these two provinces are not included into the GLOBOCAN2008 database. The NPC incidence rates in male and females of Naga, India account for 6.2 and 2.1 per 100,000 person-years, respectively [20], [21], this region is not listed within top 20 high incidence rate regions probably because of the developing cancer entry system there.

Our findings can not reflect the fact that the high NPC incidence rates stick to high risk population did not change even after they migrate to lower-risk countries. Indeed, among southern Chinese living in Singapore, Malaysia, and Japan, NPC rates are comparable with those in natives of southern China [1], [22], [23]. Likewise, NPC incidence is higher in North African migrants to Israel and their offspring than in native Israelis [24]. The inter country comparison of NPC incidence rate between different ethnics groups from the GLOBOCAN2008 database could not be performed because of the limited cancer registries.

### 2. What can be learned from the current study?

#### 2.1. The usability of this analysis for different cancer

Although the combined analysis on CIN databases (CI5, GLOBOCAN and WHO) has been succeeded in giving an international view on some specific cancer type [5], it may not be much usable for a cancer with specific ethnic/geographic distributions at the current version, especially when it is specific for the economically transition countries/regions. First, the cancer entry systems in the economically transition countries/regions are mostly under developing and may not cover all the cancers and sub regions, thus the output database quality might be low. Second, if a cancer that is restricted to a specific population or specific region, it may not appeal to the international interest for cancer research and therapy. This attitude may bias the enrolled data of this specific cancer.

#### 2.2 More effort should be put in developing the cancer entry system all over the world to offer detailed and accurate data for most cancers

Cancer with specific ethnic/geographic distribution may not affect most population all over the world, but it is a real devastating tragedy for those being affected who can be the major population of the specific region or ethnics. Thus, it has very important meaning to understand the epidemiology of the specific cancer. To offer more useful information, lots of effort should be put in developing the regional cancer entry system, especially in the economically transition countries.

Actually, CIN keeps offering efforts to help local healthy agencies improve the cancer entry system in order to finally increase the worldwide coverage of cancer registry. These activities include providing administrative facilities and a secretariat to the International Association of Cancer Registries (IACR, www.iacr.com.fr) and to the European Network of Cancer Registries (ENCR, www.encr.com.fr). CIN staffs are involved in conducting site visits to assess the feasibility of establishing new cancer registries and to provide developmental advice to existing registries, particularly in low and middle income countries. CIN arranges training courses on cancer registration and its application to epidemiology. CIN has also developed the CanReg5 software package, now used in more than 50 countries, and provides training in its use. Alongside the above activities, CIN conducts a program of research focused on descriptive epidemiology. Core components include the study of temporal trends and patterns in the occurrence of and outcomes from cancer, as well as the development of methodological approaches to their analysis.

Combining the efforts from CIN and local healthy agencies, high quality CIN databases are expected that more reliable analysis of international tendencies of most cancer types can be performed.

## Supporting Information

Figure S1 Regional incidence of pharyngeal cancer during 1998-2002 demonstrated as ASR per 100,000 person years from CI5 database (ICD 10 code: C09-14) of male (left) and female (right) populations.
(TIF)
