# Supplementary figures and images for: Can Global Variation of Nasopharynx Cancer Be Retrieved from the Combined Analyses of IARC Cancer Information (CIN) Databases?
Source: PLoS One. 2011 Jul 7;6(7):e22039. doi: 10.1371/journal.pone.0022039 (PMC3131403; doi:10.1371/journal.pone.0022039)

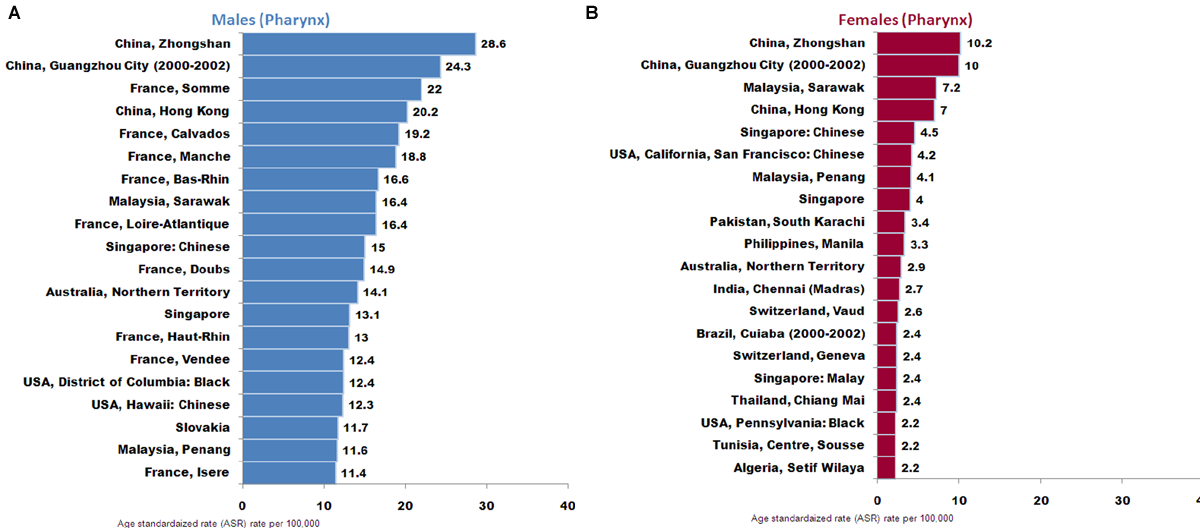

Supplement: Figure S1 — Regional incidence of pharyngeal cancer during 1998-2002 demonstrated as ASR per 100,000 person years from CI5 database (ICD 10 code: C09-14) of male (left) and female (right) populations. (TIF) [file pone.0022039.s001.tif]
